# Perception during double-step saccades

**DOI:** 10.1038/s41598-017-18554-w

**Published:** 2018-01-10

**Authors:** E. Zimmermann, M. C. Morrone, P. Binda

**Affiliations:** 1 Institute for Experimental Psychology, Heinrich Heine University Düsseldorf, Universitätsstraße 1, 40225 Düsseldorf, Germany; 2 University of Pisa, Department of Translational Research on New Technologies in Medicine and Surgery, Pisa, Italy; 3 IRCCS Stella Maris, Calambrone, Pisa, Italy; 4 CNR Neuroscience Institute, Pisa, Italy

## Abstract

How the visual system achieves perceptual stability across saccadic eye movements is a long-standing question in neuroscience. It has been proposed that an efference copy informs vision about upcoming saccades, and this might lead to shifting spatial coordinates and suppressing image motion. Here we ask whether these two aspects of visual stability are interdependent or may be dissociated under special conditions. We study a memory-guided double-step saccade task, where two saccades are executed in quick succession. Previous studies have led to the hypothesis that in this paradigm the two saccades are planned in parallel, with a single efference copy signal generated at the start of the double-step sequence, i.e. before the first saccade. In line with this hypothesis, we find that visual stability is impaired during the second saccade, which is consistent with (accurate) efference copy information being unavailable during the second saccade. However, we find that saccadic suppression is normal during the second saccade. Thus, the second saccade of a double-step sequence instantiates a dissociation between visual stability and saccadic suppression: stability is impaired even though suppression is strong.

## Introduction

During saccadic eye movements, the visual scene sweeps across the retina with high-speed motion. Yet we do not perceive very fast disturbing motion or jumps of the visual scene. What mechanisms ensure the smooth and stable perception that we experience is still an open question. It is generally agreed that the problem can be subdivided into two components: to counteract the retinal displacement and to eliminate spurious intra-saccadic motion signals.

The first problem has been connected with the neurophysiological finding of receptive field remapping: neurons in parietal^1^, frontal^2,3^ and even in early visual areas^4,5^ shift their receptive field before the saccade, to that location the receptive field will cover after the saccade, effectively counteracting the spatial displacement of the retinal image. In previous work, we have suggested that this predictive receptive field shifts may facilitate trans-saccadic vision of objects^6,7^ and are consistent with the peri-saccadic mislocalization observed psychophysically. Others have interpreted receptive field shifts as remapping of attentional pointers: movement of attention across spatial maps, rather than updating of the maps themselves^8,9^. In either case, an active mechanism that anticipates the consequences of saccades and accounts for them is required. This can be modeled as an “efference copy” signal, i.e. a copy of the motor command that drives the eye movement. A possible neural implementation of this mechanism has been identified in a signal originating in the superior colliculus, and sent through the mediodorsal nucleus of the thalamus to the frontal eye fields^10^, for lesions of this pathway lead to misperceiving intra-saccadic target displacements^11,12^.

The second problem, eliminating the motion of retinal images, probably combines several factors: both passive and active mechanisms. Among the passive mechanisms, there is the high-speed motion of the eye itself, which produces retinal smear, making the fine details in our visual scene effectively invisible. However, not all stimuli are invisible during saccades – in fact, motion that is too fast to be resolve may become visible only during the saccade, if the movement of the retina counteracts and effectively slows down the motion of the stimulus^13,14^. Another passive factor is masking: the blurred intra-saccadic image can be masked by high contrast images acquired before and after the saccade^15^. However, when all these factors are controlled for (the stimulus is a grating parallel to the saccade, minimizing blur; the stimulus is flashed, preceded and followed by a blank field, which is a very weak mask), sensitivity is still reduced. Suppression is the strongest for low spatial frequency luminance modulations^16–20^ – but never complete, amounting to about 0.5–1 log units, homogeneous across the visual field^21^ – and suppression starts *before* the saccade onset. Some have suggested that these features may still be accounted for by passive mechanisms alone. For example, Castet *et al*.^22^ have suggested that the Stiles-Crowford effect might be the key: photoreceptors bend during the saccade producing a luminance change that could, in principle, through light adaptation mechanisms, affect perisaccadic sensitivity. However, it is unlikely that this phenomenon is strong enough to explain the 0.5–1 log units drop of sensitivity; and if it were, it is unclear why such large luminance change would itself remain invisible^23^. On the other hand, suppression might be achieved through an active mechanism, perhaps relying on a similar efference copy signal as the one mediating spatial stability^19,24^. Although much research has attempted to identify the neural underpinnings of such mechanism, these are still unclear^25–27^.

Here we aim to contribute to these discussions by asking whether perisaccadic remapping and suppression are driven by the same process. We approach this problem by studying a condition where saccade planning (and the accompanying efference copy) may be partly dissociated from saccade execution: the double-step saccade paradigm^28,29^. In this paradigm, two targets are presented briefly, extinguished, and subjects are asked to saccade to them sequentially. The interval between end of the first and start of the second saccade can be extremely short (down to 20 ms), suggesting that they are planned in parallel^30^. Pre-planning of the second saccade is further supported by an inverse relationship between the latency of the first saccade, and the inter-saccadic interval between the two^30,31^. In addition, Zingale and Kowler^32^ showed that saccade parameters like latency and duration, co-vary with the total amplitude of the saccade sequence, rather than depending on the individual amplitudes, implying that all saccades in the sequence are globally planned and stored as a package rather than serially.

We reasoned that, if most saccade planning is completed before execution of the first saccade, then any phenomenon that is dependent upon efference copy information may be impaired during the second saccade. We therefore asked whether the second saccade is accompanied by an impairment of either or both the two key aspects of perisaccadic visual perception described above: spatial stability and contrast suppression.

Spatial stability was tested using trans-saccadic apparent motion^33,34^. When two stimuli are presented asynchronously and orthogonally to the saccade path, one before and one after the saccade, their apparent motion trajectory can only appear vertical if the movement of the eyes is perfectly accounted for. On the other hand, if the motion trajectory is slanted, under- (or over-) compensation of the eye movement is implied – a partial failure of spatial stability. This phenomenon cannot be explained by perisaccadic mislocalization of flashed visual stimuli^33,34^, but the two phenomena may be related in that they may both depend upon efference copy information. We have recently investigated perisaccadic mislocalization in the context of a double-step saccade task, and found that it is much reduced during the second saccade of the sequence^31^. Here we test whether apparent motion trajectories also differ between the second saccade of a double-step sequence and a single saccade of matched amplitude and trajectory.

Using the same double-step paradigm, we also tested perisaccadic suppression, measuring sensitivity to a 0.1 cycles per degree grating, shown parallel to the saccade (to minimize retinal smear) for one monitor frame. By varying the time of stimulus presentation, we determined contrast sensitivity over the full time-course of the double-step saccade sequence as well as during single saccades with matched amplitudes.

## Results

### Double-step saccade performance

**Figure 1:**
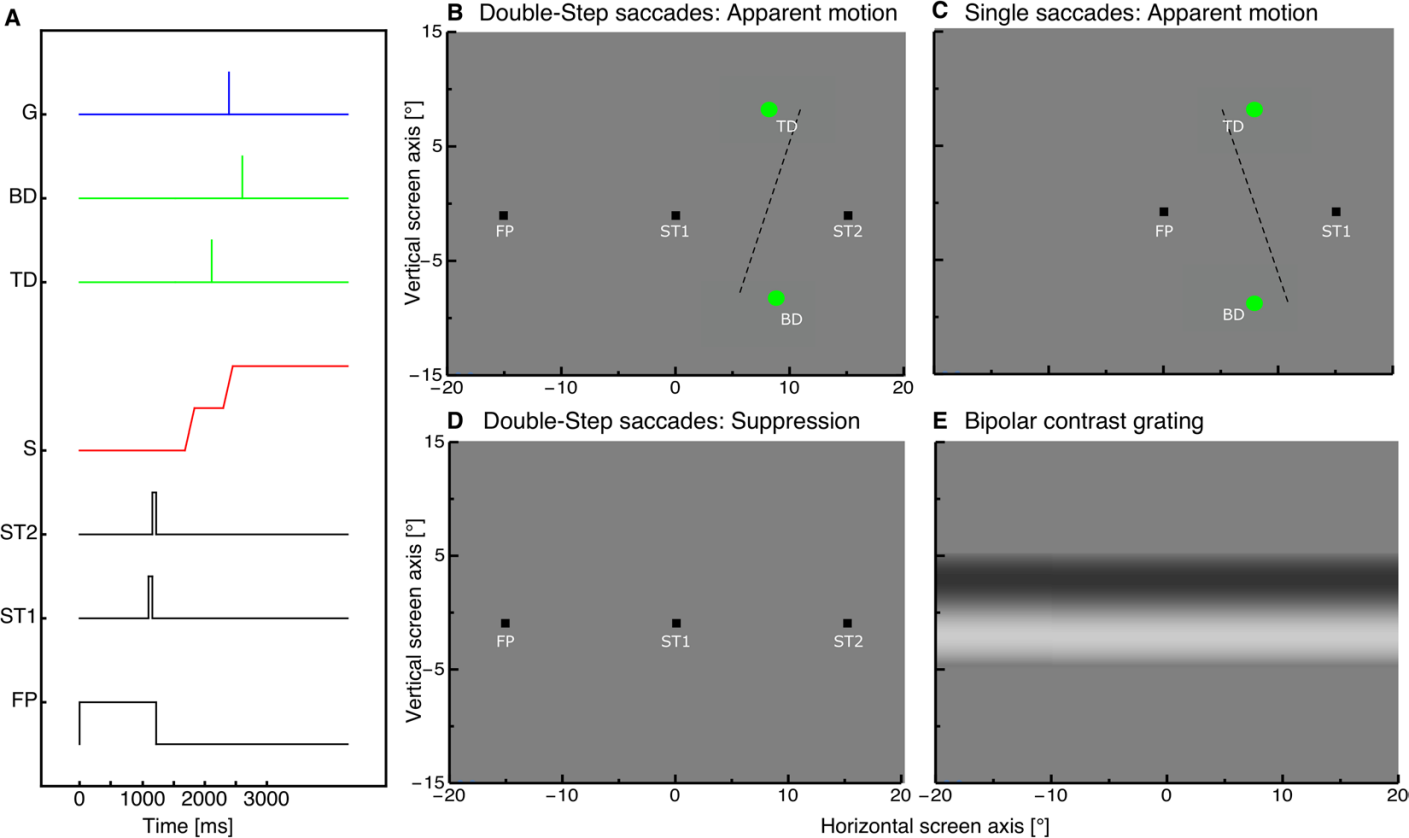
(**A**) Timecourse of presentations. Subjects made double-step saccades from a fixation point (FP) to the first saccadic target (ST1), and from there to a second saccade target (ST2). In experiment 1, two dots were shown around the time of the second saccade, in the top (Top Dot, TD) and bottom half of the screen (Bottom Dot, BD), both for one monitor frame. In experiment 2, a grating (G) was shown for one monitor frame at a variable time, before, during or after the double-step saccade sequence. (**B**) Spatial arrangement of the stimuli in experiment 1, where apparent motion was perceived between two dots shown across the second saccade of the double-step sequence. The dashed line (not part of the experimental display) indicates the direction of apparent motion that subjects typically reported in this condition, for dots that were physically aligned: motion was slightly slanted to the right, indicating under-compensation of the saccade vector. (**C**) Control condition of experiment 1, where the apparent motion stimulus was shown across a single saccade between ST1 and ST2. In this condition, the direction of apparent motion was typically slanted to the left, indicating over-compensation of the saccade vector, consistent with previous studies (Szinte and Cavanagh^34^). (**D**–**E**) Spatial arrangement of the stimuli in experiment 2, with fixation and saccade targets in panel D and the contrast discrimination stimulus in (**E**) (the latter could be shown with this or the opposite polarity, at variable contrast levels).

**Figure 2:**
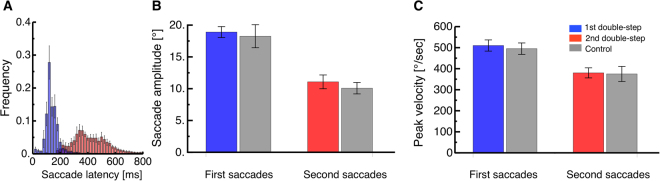
(**A**) Average frequency distribution of saccade latencies for the first (blue) and second (red) saccades in the double-step sequence. Histograms are computed in individual subjects then averaged across subjects, with error bars representing SEM. (**B-C**) Average saccade amplitudes (**B**) and peak velocities (**C**) for the first (blue) and second saccades (red) in the double-step saccade paradigm and for the single saccades in the control condition (shown in gray). Error bars represent SEM.

In both our Experiments 1 and 2, observers performed a sequence of saccades to two successively presented targets in a classical double-step paradigm (see Fig. 1). There were no systematic differences between saccades from the first and the second Experiment, implying that the presentation of the perceptual stimulus (apparent motion or grating) did not affect saccade performance. We therefore computed saccade parameters after pooling across experiments. Subjects had to initiate their saccades as soon as the fixation point disappeared. Figure 2A shows the distribution of saccade latencies for the first (blue) and second (red) saccade in the double-step sequence (histograms were computed for each subject then averaged across all subjects. Latencies averaged 130.42 ms (SEM: 14.04) and 418.18 ms (SEM: 42.01) for the first and second saccade respectively. Inter-saccadic intervals could be extremely short, with a minimum of 21 ms; in 11% of trials (SEM 5%) they were below 80 ms, although the average was 218.10 ms (SEM 19.52 ms). These figures are consistent with prior measurements [30] and the parallel planning hypothesis they inspired.

Amplitude also differed for the first and second saccade (Fig. 2B) although the required saccadic amplitude was 15° in both cases: first saccades (blue, average: 19.20°, SEM: 0.98) were larger than second saccades (red, average: 11 °, SEM: 1.12; paired t-test of amplitude of the first vs. second saccade: t(5) = 6.633, p = 0.001). This is again in line with earlier studies, where the landing position of the first saccade often deviated in direction of the second saccade target^30^. Similarly, peak velocities (Fig. 2C) were higher for the first saccade (red, average: 503.60 °/sec, SEM: 32.99 °/sec) than for the second saccade (blue, average: 369.49 °/sec, SEM: 28.82 °/sec). Gray bars in Fig. 2B–C give the parameters of saccades in the control condition, where the positions of ST1 and ST2 were adjusted so that the start and end-position of the single saccades matched those of the first or second saccade of the double-step sequence. As a result, the amplitude and peak velocity of the single saccades matched those of the first or second saccade of the double-step sequence.

### Experiment 1: Apparent motion

With an apparent motion display, presented during the double-step sequence or the control-single saccades, we tested whether saccade preplanning modulates spatial stability. The apparent motion stimuli were presented (on average) at the same horizontal screen position, below and above the saccade path (see Fig. 1B). However, they were presented across the saccade (the second saccade of the double-step sequence, or the single saccade in the control condition): one before saccade onset, the other after saccade completion, so that the saccade dissociates retinal from screen coordinates. In order to perceive vertical apparent motion in screen coordinates, the saccade vector must be compensated for – likely through efference copy information.

**Figure 3:**
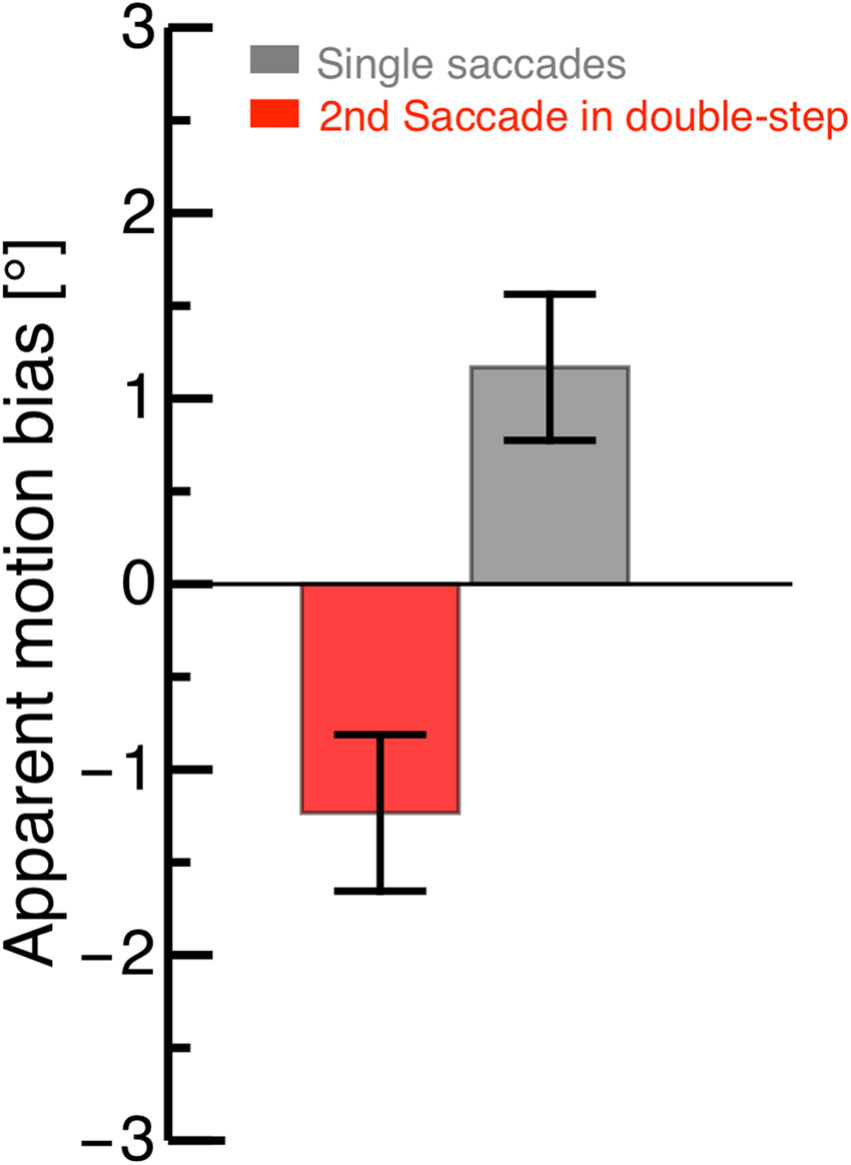
Average apparent motion bias for stimuli presented across the second saccade of the double-step sequence (red) and across a single saccade in the control condition (gray). Error bars represent SEM. A positive/negative bias implies a bias towards seeing rightward/leftward motion (illustrated by the dashed lines in Fig. 1B/C). A negative bias is consistent with a slight overcompensation of the saccade vector.

Previous studies have shown that apparent motion is indeed seen as approximately vertical, though a systematic slant of motion trajectories implies a tendency to overcompensate for the saccade vector^34^. We confirm this result in the control condition, where the apparent motion display was shown across a single saccade (Fig. 3 gray bar; see also dashed line in Fig. 1C). However, the results were very different when we presented the apparent motion stimuli across the second saccade of the double-step sequence. Apparent motion trajectories were slanted in the opposite direction (Fig. 3 red bar; see also dashed line in Fig. 1B), implying that the saccade vector was under-compensated. This is consistent with the hypothesis that efference copy information is impaired at the time of the second saccade of a double-step sequence, the calibration being largely carried out once and for all at the beginning of the sequence, i.e. at the time of the first saccade.

We found a significant difference between apparent motion judgments during the second saccade of a double-step sequence and those tested during a single saccade (Mann-Whitney-U-test: p = 0.01). We verified that the difference between the double-step condition and the single-saccade condition cannot be explained by differences in the characteristics of saccades, which where statistically indistinguishable (paired t-tests on amplitude: t(3) = 0.31, p = 0.78; peak velocity: t(3) = 1.86, p = 0.16). One theoretical possibility is that the apparent motion biases are explained out by perisaccadic mislocalization, another saccade-related phenomenon also known to differ between single and double-step saccades^31^. However, two considerations argue against this hypothesis. First, we only analysed trials where neither of the apparent motion stimuli fall into the temporal window of perisaccadic mislocalization (extending some 50ms around each saccade). Second, we performed an additional analysis comparing the results from two sub-samples of the single-saccade control trials: where the first apparent motion stimulus appeared more than 75 ms before the saccade (mislocalization unlikely but possible) or more than 150 ms before the saccade (no mislocalization). Apparent motion biases were very similar: 1.21 (SEM 0.48) and 1.17 (SEM 0.39). We are thus confident that perisaccadic mislocalization does not interfere with the discrimination of the apparent motion slant.

### Experiment 2: Contrast Thresholds

**Figure 4:**
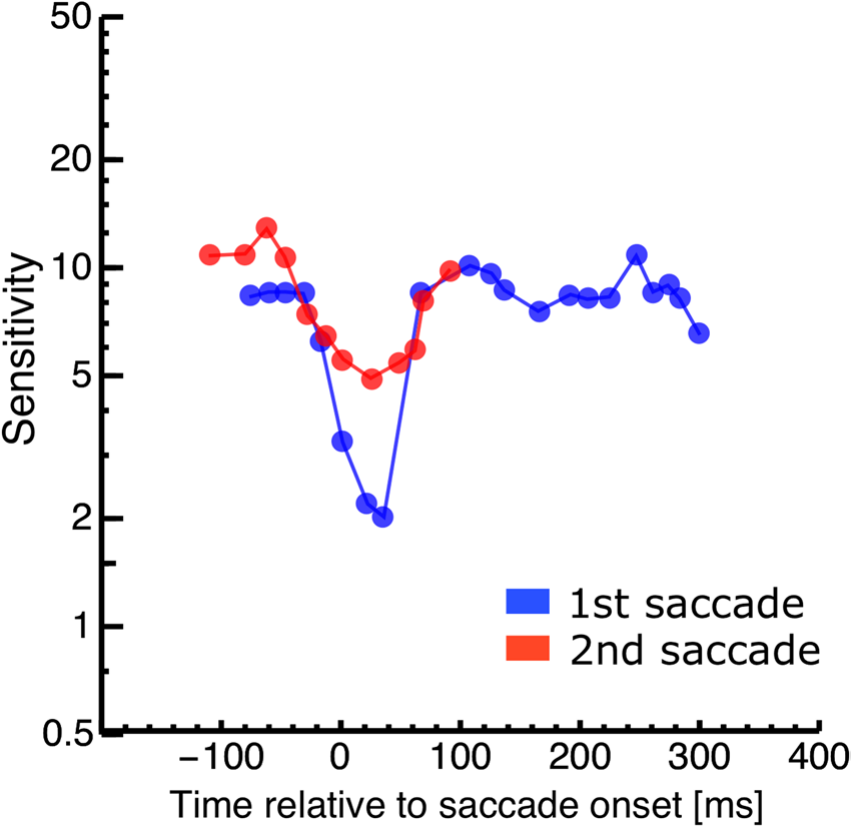
Contrast sensitivity as a function of probe presentation time relative to saccade onset for a representative subject. Data are binned relative to onset of the first saccade (shown in blue) or relative to onset of the second saccade (shown in red).

**Figure 5:**
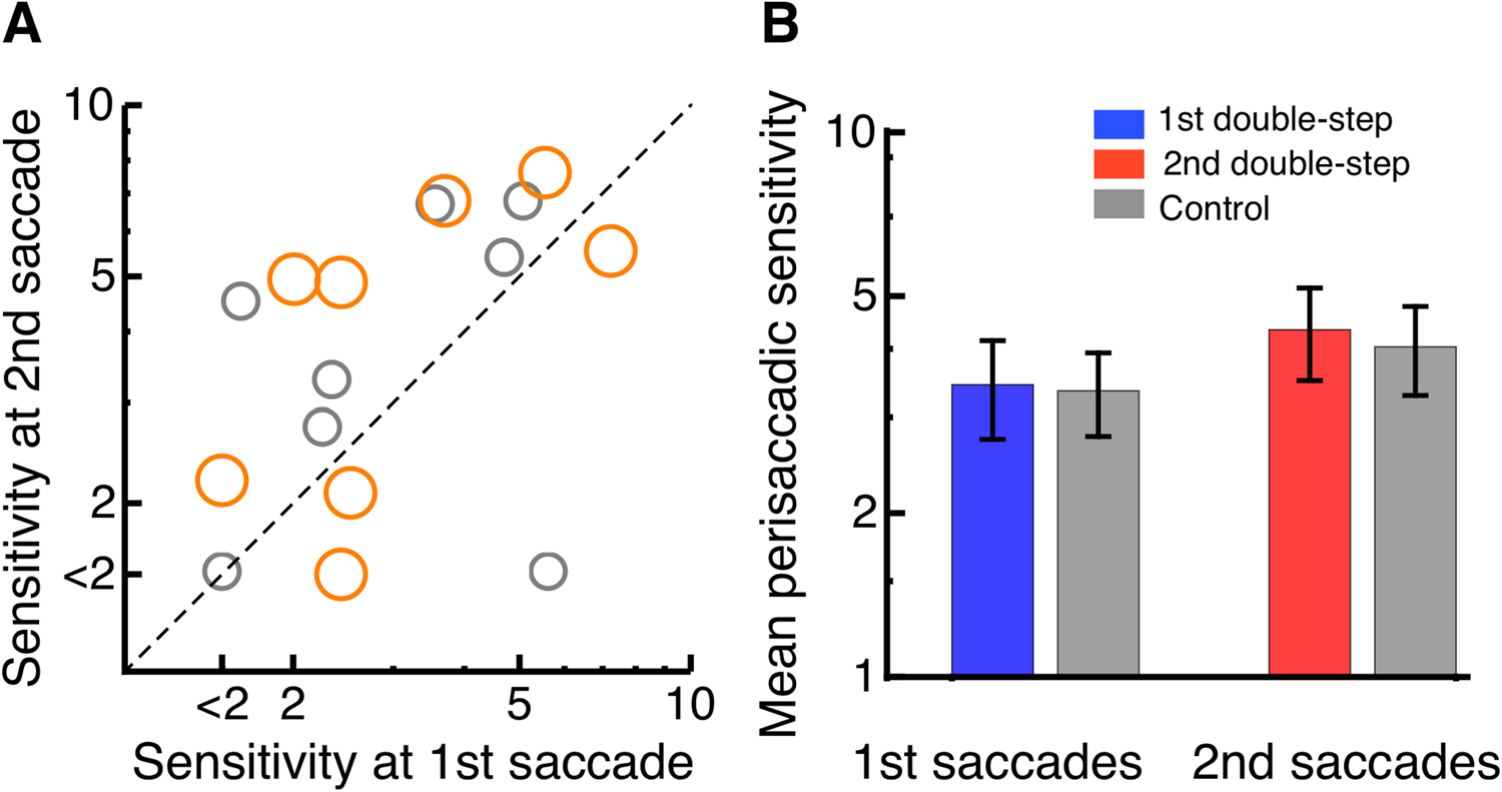
(**A**) Orange points give perisaccadic sensitivity during the second (ordinate) vs. the first (abscissa) saccade in a double-step sequence, and gray points give sensitivities for the control condition, with single saccades of amplitude matched to the second or first saccade of the sequence respectively. Each point is one subject. Maximal contrast in our setup was 0.5, thus sensitivity below 2 was not measurable and is indicated in the figure by the tick mark “<2”. (**B**) Average perisaccadic sensitivity during the first and second saccade of the double-step sequence (red and blue respectively) and during single-saccades of matched amplitude (gray) Error bars represent SEM.

We next asked whether perisaccadic suppression is also atypical during the second saccade in the double-step paradigm. We tested sensitivity to a low-spatial frequency grating shown parallel to the saccade path, for one monitor frame. The stimulus could appear around the time of either saccade of the double-step sequence, or during a single saccade of matched amplitude and trajectory (see Fig. 1A,D,E). Figure 4 shows the time-course of contrast sensitivity for one subject. Contrast sensitivity aligned to the onset of the first saccade is shown in blue and sensitivity aligned to the second saccade in red. Suppression occurs during both saccades: contrast sensitivity decreases for gratings presented near the saccade onset and with a minimum at saccade onset and a recovery within 100 ms from the saccade. Sensitivity reaches a lower minimum during the first than the second saccade (2 vs. 5), implying weaker suppression during the second than the first saccade. This was consistently seen across subjects: Fig. 5A shows results from all subjects, plotting minimum sensitivity during the second saccade (ordinate of orange dots) vs. minimum sensitivity during the first saccade (abscissa or orange dots). Colored bars in Fig. 5B give the average values across all subjects. We had found previously a reduction of peri-saccadic mislocalization during the second compared with the first saccade in the double-step paradigm. We therefore compared peri saccadic contrast sensitivity between the first and the second saccade. However, the difference between minimum sensitivity during the first and the second saccade was not significant (paired t-test, t = −1.16, p = 0.14). (Maximal contrast in our setup was 0.5, thus sensitivity below 2 was not measurable. One subject with a sensitivity below 2 was excluded from the ttest statistic). This pattern of results, however, is not a peculiarity of the double-step saccade paradigm, but a simple consequence of the amplitude disparity between the first and second saccade – given that suppression is stronger for larger saccades^35,36^. Indeed, a similar difference of minimum perisaccadic sensitivity could be reproduced in the single saccade paradigm (gray in Fig. 5). We calculated a Bayes factor^37^ to test the differences in sensitivity between first and second saccade of the double-step and first and second saccade of the single-step paradigm. The analysis estimated a Scaled JZS Bayes Factor, that combines the Cauchy distribution on effect size and the Jeffreys prior on variance. The analysis revealed a Scaled JZS Bayes Factor of 2.79 in favor of the null hypothesis, which can be considered as substantial evidence^38^.

## Discussion

We tested trans-saccadic stability and perisaccadic suppression during double-step saccades. The double-step paradigm is designed to dissociate saccade planning from saccade execution factors^39^. Since both saccade targets are extinguished before the eye starts to move, the sensorimotor system cannot rely on visual guidance for saccade execution. It has been suggested that the system preplans both saccades in advance^30^. The idea of preplanning was supported by very short temporal interval durations between the first and the second saccade which can be as short as 20 ms, far less than the 125 ms (minimum) required for programming of single saccades^30,40^. We observed intersaccadic interval durations as short as 21 ms and 10% of all trials were far below the time that saccade planning usually takes. Even saccades with the shortest latency, i.e. express saccades have a latency of at least 100–120 ms in humans^41^.

We investigated whether saccade planning affects the trans-saccadic perception of visual space. Electrophysiological work has demonstrated that receptive fields shift shortly before saccade initiation to compensate for the retinal displacement^1^. We reasoned that, if planning of the second saccade in the double-step paradigm occurred already before the first saccade, then compensation for the gaze displacement could be impaired at the time of the second saccade. We used the paradigm of trans-saccadic apparent motion that is well suited to test how gaze displacement is accounted for in the perception of space^33,34,42^. In this paradigm, one stimulus is shown before and another after saccade execution. Together these two stimuli produce the perception of apparent motion. If stimuli are placed such that the apparent motion path is orthogonal to the saccade path, then the orientation of the apparent motion path indicates how the saccade vector is integrated into vision. Szinte and Cavanagh^34^, in single saccades, found a slight over-compensation of the saccade vector leading to apparent motion slightly tilted against the direction of the saccade. We confirm this result for single saccades. However, apparent motion across the second saccade of the double-step sequence was consistent with under-compensation of the saccade vector. This is consistent with the idea that part of the remapping process for the second saccade had already been accomplished before first saccade execution.

Our results provide indications that this bias in apparent motion is not explained out by perisaccadic mislocalization, the erroneous localization for briefly flashed stimuli presented within few tens of milliseconds from the saccade onset^43–46^. Yet, we do believe that compensation of the saccade vector (by means of efference copy) is involved in both apparent motion and flash localization, and that the two phenomena are closely linked. Our recent work^31^ showed that perisaccadic compression of visual space is strongly reduced during the second saccade in a double-step sequence. Here we find that apparent motion perception is altered at the same time. This is consistent with both phenomena resulting from a process that restores visual stability across saccades^7^.

Perisaccadic suppression of contrast sensitivity, however, proved to behave in a very different way. We found that suppression is equally strong during the second saccade of a double-step saccade sequence, and during any single saccade with similar amplitude and velocity. Suppression was weaker during the second than the first saccade of the double-step sequence, but the difference was easily accounted for by the different amplitude of the two saccades – for, consistent with earlier reports^30^, subjects generated larger first and smaller second saccades, and there is well established monotonically increasing relationship between the strength of perisaccadic suppression and saccade amplitude^36^.

To summarize, we find that saccadic suppression is unaltered during the second saccade of a double-step sequence, whereas at the same time both apparent motion judgments (measured here) and localization judgments^31^ are atypical.

Based on these findings, we speculate that the organization of space perception across saccades, which affects both apparent motion and localization judgments, is linked to saccade planning: it is impaired when planning is dissociated from saccade execution, i.e. in the double-step paradigm. Saccadic suppression, which is insensitive to this manipulation, appears to be linked to saccadic execution instead.

This does not necessarily imply that saccadic suppression is independent of efference copy information. As reviewed in the Introduction, many prior studies yielded results that are difficult to account for with passive mechanisms alone, thus supporting the hypothesis of an active suppression driven by efference copy information. Thus, the dissociation we observe can be interpreted by assuming that suppression and remapping depend on independent active processes: there could be two independent sites where efference copy signals interact with visual signals. According to one theory^18^, the interaction should occur at an early site for perisaccadic suppression, which may be achieved already in the thalamus^24–26^. Organizing visual space, on the other hand, requires integrating multiple sources of information, including visual references available before and after saccades (as already discussed^41^). Such complex integration may happen at a later stage of visual processing, making it more sensitive to manipulations such as the one employed here.

## Materials and Methods

### Participants

Four observers (1 male, 3 female, mean age: 30 years) participated in Experiment 1. The same four observers plus four different observers (4 male, 4 female, mean age: 29 years) participated in Experiment 2. All subjects had normal or corrected-to-normal vision. Subjects gave informed consent. Experimental procedures were approved by the regional ethics committee [Comitato Etico Pediatrico Regionale—Azienda Ospedaliero-Universitaria Meyer—Firenze (FI)]. Written informed consent was obtained prior to each experiment in accordance with the declaration of Helsinki.

### Apparatus

Subjects sat 57 cm from a 22 inch CRT color monitor (Barco Calibrator, refresh rate: 120 Hz; Resolution: 800 × 600 pixels corresponding to a 40° × 30° field of view) displaying a uniform gray field of 13 cd/m2.

### Eye movements and data analysis

Eye movements were monitored by the EyeLink 1000 system (SR Research), which samples gaze positions with a frequency of 1000 Hz. Viewing was binocular, but only the dominant eye was recorded. A standard 9 point calibration was performed at the beginning of each block of trials. The system detected the start and the end of a saccade when eye velocity exceeded or fell below 22 °/s and acceleration was above or below 4000 °/s^2^. In the offline analysis, we checked these estimates (and, when a double-step saccade sequence was required, we excluded trials in which only one saccade could be detected). In both experiments, trials were rejected if only one saccade was performed, if the first or second saccade was smaller than 5° or if the first saccade started before offset of the fixation point. In Experiment 1 on average 591 and in Experiment 2 on average 990 trials were collected.

### Saccade task

Trials for both experiments started with the presentation of a black fixation point (0.75 × 0.75°) 15° to the left of screen center. After 1000 ms plus a randomly chosen duration between 0–500 ms, the first saccade target ST1 (0.75 × 0.75°, black) appeared at screen center (see Fig. 1A,B), which remained visible for 64 ms. Upon extinction of ST1, ST2 appeared at 15° to the right of screen center and remained visible for another 64 ms. ST2 and the fixation point were extinguished together and this cued subjects to start the saccade sequence: from the fixation point to ST1, and from ST1 to ST2. In a control condition, subjects executed a single saccade between FP and ST1 or between ST1 and ST2. We first analysed amplitudes from the double-step saccade blocks. We then placed the targets ST1 and ST2 so to match the amplitude of the first or the second saccade in the double-step sequence. In these single saccade trials, the first target was shown for 1000 ms plus a randomly chosen duration between 0–500 ms. After it went off, the second target appeared and subjects saccaded to it. All conditions (double-step, control: first saccade, control: second saccade) were run in separate blocks.

We estimated for each participant the average time of the second saccade based on the analysis of pilot trials. We used these data to present the apparent motion stimulus around the time the saccade to target ST2 would be performed (Fig. 1B). At about the time of the saccade to ST2, the apparent motion stimulus was delivered (Fig. 1B). This consisted of two green dots (1.5° diameter), each flashed for one monitor frame (8 ms) with a temporal separation of 356 ms on average (which varied slightly across subjects to optimize data collection, see below). The first dot was shown above gaze level (Top Dot, TD, y = 10°) at a fixed horizontal location (halfway between ST1 and ST2, x = 7.5°). The second dot was shown below gaze level (Bottom Dot, BD y = −10°) at variable horizontal location (x = 7.5° ± 3°). The stimulus was perceived as a single dot moving downward with a near-vertical trajectory, i.e. orthogonal to the direction of saccades. We only analyzed trials where the ST1-ST2 saccade occurred between the two dots (first dot at least 50 ms before the saccade, second dot after its completion); therefore, in retinotopic coordinates, they were always displaced horizontally by about 15°. That subjects perceived them as moving along a nearly vertical trajectory, indicates that the retinotopic displacement is largely compensated, ensuring spatial stability. However, small biases of motion direction can indicate relative failures in this stabilization process. To estimate these, we varied the location of the second (bottom) dot with the method of constant stimuli and asked subjects to report in 2AFC whether the motion trajectory was slanted to the right or to the left relative to the vertical (implying a component of rightward motion or leftward motion). Data were analyzed as psychometric curves, plotting the proportion of “rightward motion” judgments (bottom dot to the right of the top dot) as function of the position of the bottom dot relative to the top dot. Distributions were fit with cumulative Gaussian functions; the median of the curve estimated the PSE, or the position of the bottom dot that led to vertical apparent motion. A negative bias (PSE < 0) implies a bias towards seeing rightward motion (as in Fig. 1C, dashed line) and a positive bias (PSE > 0) implies a bias towards seeing leftward motion (as in Fig. 1C, dashed line). The former (negative bias) is consistent with Szinte and Cavanagh^34^ and can be interpreted as a slight overcompensation of the saccade vector.

### Experiment 2: Contrast Thresholds

At a randomly chosen time between 50–500 ms starting from fixation point offset, a horizontally oriented sinusoidal grating was shown for 8 ms (“G” in Fig. 1A, Fig. 1E). The grating was a cosine function of 0.1 cycles/° masked by a vertically oriented Gaussian envelope with 10° space constant. The stimulus was symmetric around the horizontal midline of the monitor, and could be shown with phase of either 0 or 180 degrees (opposite polarity, varied randomly across trials). The contrast of the stimulus was varied across trials between the maximum and minimum values attainable in our set up (0.0079 and 0.5023 Michelson contrast). Subjects reported the stimulus polarity it in 2AFC by pressing the arrow-keys on the computer keyboard at the end of each trial. During the response interval the screen remained blank. The response was not speeded and the next trial started when participants pressed one of the arrow-keys. Trials were binned per time of stimulus presentation relative to the onset of the first saccade, or the second saccade in the double-step sequence (or relative to the onset of the single saccade in the control condition). Data were analyzed as psychometric curves, plotting percent correct against stimulus contrast and fitting the distribution with a cumulative gaussian function (varying between 50% and 100% correct); the median of the fit estimated the visibility threshold and sensitivity was computed as the inverse of this value.

### Ethical statement

Written informed consent was obtained prior to each experiment in accordance with the Declaration of Helsinki. Experimental procedures were approved by the regional ethics committee [Comitato Etico Pediatrico Regionale—Azienda Ospedaliero-Universitaria Meyer—Firenze (FI)] and conducted in accordance with their guideline.
